# Hsa-miR-3651 could serve as a novel predictor for in-breast recurrence via FRMD3

**DOI:** 10.1007/s12282-021-01308-y

**Published:** 2021-12-05

**Authors:** Barbara Zellinger, Ulrich Bodenhofer, Immanuela A. Engländer, Cornelia Kronberger, Brane Grambozov, Elvis Ruznic, Markus Stana, Josef Karner, Gerd Fastner, Karl Sotlar, Felix Sedlmayer, Franz Zehentmayr

**Affiliations:** 1 Department of Radiation Oncology, Paracelsus Medical University, SALK, Müllner Hauptstrasse 48, 5020 Salzburg, Austria; 2 radART—Institute for Research and Development on Advanced Radiation Technologies, Paracelsus Medical University, Salzburg, Austria; 3 Department of Pathology, Paracelsus Medical University, SALK, Salzburg, Austria; 4 School of Informatics, Communications and Media, University of Applied Sciences Upper Austria, Hagenberg, Austria; 5 Institute for Machine Learning, Johannes Kepler University, Linz, Austria

**Keywords:** Hsa-miR-3651, FRMD3, Early breast cancer, Local control, Prediction

## Abstract

**Background:**

MicroRNAs are small non-coding RNAs with pivotal regulatory functions in multiple cellular processes. Their significance as molecular predictors for breast cancer was demonstrated in the past 15 years. The aim of this study was to elucidate the role of hsa-miR-3651 for predicting of local control (LC) in early breast cancer.

**Results:**

By means of high-throughput technology, hsa-miR-3651 was found to be differentially expressed between patients who experienced local relapse compared to those without (*N*  =  23; *p*  =  0.0035). This result could be validated in an independent cohort of 87 patients using RT-qPCR (*p*  <  0.0005). In a second analysis step with a chip-based microarray containing 70,523 probes of potential target molecules, FERM domain protein 3 (FRMD3) was found to be the most down-regulated protein (*N*  =  21; *p*  =  0.0016). Computational analysis employing different prediction algorithms revealed FRMD3 as a likely downstream target of hsa-miR-3651 with an 8mer binding site between the two molecules. This could be validated in an independent patient set (*N*  =  20, *p*  =  0.134).

**Conclusion:**

The current study revealed that hsa-miR-3651 is a predictor of LC in early breast cancer via its putative target protein FRMD3. Since microRNAs interfere in multiple pathways, the results of this hypothesis generating study may contribute to the development of tailored therapies for breast cancer in the future.

**Supplementary Information:**

The online version contains supplementary material available at 10.1007/s12282-021-01308-y.

## Introduction

The breast cancer mortality rate in Europe is predicted to be 13.4 per 1,00,000 for 2020 [1]. Loco-regional control is a cornerstone for long-term cure. An analysis by the Early Breast Cancer Trialist Group (EBCTG) revealed a 4:1 relation between local relapse and breast cancer specific death [2]. Therapeutic advances in all disciplines related to breast conserving therapy (BCT) have led to low local relapse rates, with age being the most important predictor. The estimates for age adjusted annual in-breast recurrence are 0.4–0.7% for patients  > 50 years, 0.72–1.2% in the age group 41–50 years, and 0.72–2% for patients younger than 40 years, respectively [3]. As the prevention of local relapse is fundamental for long-term cancer free survival especially in younger patients [2], pre-therapeutic molecular tumor profiling is increasingly important.

MicroRNAs are small non-coding RNAs (ncRNA) of 20–25 nucleotides, which interfere in multiple (patho)physiological processes via transcriptional regulation. During the past 2 decades, they have emerged as a new class of predictive and prognostic markers in various types of cancer. As for breast cancer, Iorio suggested a miR-signature, describing a panel of de-regulated miRs correlated with known clinical and biological features such as hormonal receptor status, tumor size, lymph node status, vascular invasion, proliferation index and p53 [4]. Integrating of molecular markers in the clinical subtyping of breast cancer [5] opens up the way for ncRNA as a prediction tool in the future. The significance of microRNAs for local control (LC) could be demonstrated in three previous publications [6–8]. While hsa-miR-3651 has been described in other cancer types such as head and neck [9, 10], ovarian [11], colorectal [12], liver [13] and lung [14], the current study is the first to demonstrate its relevance in breast cancer. Thus far, no data are extant on its potential target proteins, let alone a potential pathway which it could interfere in. Computational analyses suggested FERM domain containing protein 3 (FRMD3), a member of the protein 4.1 superfamily, as a putative downstream target, which is involved in cytoskeletal protein binding and seems to play a role in cell motility and invasiveness [15–17].

The aim of the current study was to identify microRNAs that could predict in-breast recurrence in locoregionally highly invasive early stage breast cancers. This hypothesis-generating study is meant to enlarge the spectrum of tailored therapies for this disease in the future.

## Methods

### Experimental design and clinical endpoint

The first step of the current study was conducted in a pilot phase by means of high-throughput technology to screen for de-regulated microRNAs with a subsequent validation phase using real-time polymerase chain reaction (RT-qPCR, supplementary file 1). A crucial point in studies like this is the heterogeneous microRNA expression in the various tissue compartments of a sample representing the molecular difference between stroma and cancer [18]. While some analyses in FFPE material were performed after microdissection [19] in order to enhance the tumor signal, we—similar to Lyng [20]—selected patient samples with a minimum tumor content of 50%. Additionally, this investigation emphasizes the role of single miRNAs. In fact, most analyses also include clustered microRNAs, which are potentially overrepresented in the miRnome because of their enhanced transcriptional efficacy based on genomic proximity [21]. In a second step, computational research was combined with a microarray to screen for potential target proteins, which was followed by a validation phase employing droplet digital polymerase chain reaction (ddPCR). While in most studies survival is the clinical endpoint [19, 20], in the current study it is local relapse defined as the re-appearance of cancer in the same breast [22].

### Patients

The patients in this investigation constitute a sub-population characterized by enhanced loco-regional invasiveness (i.e., higher T-, N-stage and increased probability of in-situ component) compared to the main cohort [7, 8]. One-hundred and ten patients were included. Thirty-seven patients with local in-breast recurrences were matched to 73 controls without relapse, according to the following criteria: year of diagnosis, type of surgery (mastectomy or lumpectomy), type of radiotherapy (whole breast irradiation with percutaneous or intraoperative boost), age, tumor size, lymph node involvement, grading, histology, hormonal receptor status, her2 status, menopausal status, Ki67 proliferation index (Tables 1, 2, 3, 4). The miRNA pilot phase included 13 patients and 10 controls, while the remaining 87 patients (24 relapses/63 controls) constituted the validation cohort. The analysis for potential target proteins was planned to be carried out in the very same patient data set and biological material. However, due to RNA degradation in the pathologic specimen that precluded proper signal detection in some cases and forensic restrictions in others the cohort had to be reduced to 21 (13 relapses/8 controls) in the pilot phase and 20 (9 relapses/11 controls) in the validation phase.

**Table 1:** Clinical and therapeutic parameters of the patients analyzed for hsa-miR-3651

Patient and treatment characteristics *N* = 110						
Parameters	Pilot phase *N* = 23			Validation phase *N* = 87		
	Relapse *N* = 13 (%)	Control *N* = 10 (%)	*p* value	Relapse *N* = 24 (%)	Control *N* = 63 (%)	*p* value
Age at diagnosis (years)						
Median	57	54	0.784	50	52	0.658
Range	36–71	35–74		33–79	37–78	
Menopause (*N*)						
No	3 (23%)	2 (20%)	0.842	9 (38%)	29 (46%)	0.126
Yes	7 (54%)	5 (50%)		14 (58%)	26 (41%)	
Unclear	3 (23%)	3 (30%)		1 (4%)	8 (13%)	
T (*N*)						
T1	7 (54%)	4 (40%)	0.738	20 (83%)	54 (86%)	0.904
T2	6 (46%)	6 (60%)		4 (17%)	9 (14%)	
N (*N*)						
N0	11 (85%)	7 (70%)	0.832	18 (75%)	49 (78%)	0.989
N1	1 (8%)	2 (20%)		6 (25%)	17 (22%)	
N2	1 (8%)	1 (10%)		0 (0%)	0 (0%)	
M (*N*)						
M0	13 (100%)	10 (100%)	1	24 (100%)	66 (100%)	1
Grading (*N*)						
G1	0 (0%)	0 (0%)	0.784	3 (13%)	5 (8%)	0.434
G2	6 (46%)	5 (50%)		13 (54%)	44 (70%)	
G3	7 (54%)	5 (50%)		8 (33%)	14 (22%)	
Histology (*N*)						
IDC	11 (85%)	10 (100%)	0.784	17 (71%)	51 (81%)	0.246
ILC	2 (15%)	0 (0%)		4 (17%)	6 (8.5%)	
Other	0 (0%)	0 (0%)		3 (13%)	6 (8.5%)	
In situ component (*N*)						
Yes	12 (92%)	3 (30%)	0.376	14 (58%)	40 (63%)	0.569
No	1 (8%)	7 (70%)		10 (42%)	23 (37%)	
Receptors (*N*)						
ER positive	6 (46%)	6 (60%)	0.605	18 (75%)	51 (81%)	0.459
ER negative	7 (54%)	4 (40%)		6 (25%)	12 (19%)	
PR positive	7 (54%)	4 (40%)	0.446	17 (71%)	46 (73%)	0.723
PR negative	6 (46%)	6 (60%)		7 (29%)	17 (27%)	
her2neu (*N*)						
Positive	8 (62%)	3 (30%)	0.208	9 (38%)	18 (29%)	0.725
Negative	5 (38%)	7 (70%)		14 (58%)	33 (52%)	
Not assessable	0 (0%)	0 (0%)		1 (4%)	12 (18%)	
Proliferation index (*N*)						
ki67 < 20%	8 (62%)	3 (30%)	0.208	14 (58%)	46 (73%)	0.179
ki67 > 20%	5 (38%)	7 (70%)		10 (42%)	17 (27%)	
Not assessable	0 (0%)	0 (0%)		0 (0%)	0 (0%)	
Boost						
Intraoperative (*N*)	7 (54%)	5 (50%)	0.879	10 (42%)	24 (38%)	0.996
Percutaneous (*N*)	6 (46%)	5 (50%)		14 (58%)	39 (62%)	
None	0 (0%)	0 (0%)		0 (0%)	0 (0%)	
Intraoperative dose (Gy)	10	10		10	10	
Percutaneous dose (Gy)	12	12		12	12	
WBRT dose (Gy)						
Median	54	54	0.648	54	54	0.571
Range	52.2–55.6	51–56		51–59	52.2–57	
Surgery (*N*)						
BCT	13 (100%)	10 (100%)	0.693	24 (100%)	63 (100%)	1
Mastectomy	0 (0%)	0 (0%)		0 (0%)	0 0%)	
Re-excision (*N*)						
Yes	5 (38%)	1 (10%)	0.483	10 (42%)	27 (43%)	0.8
No	8 (62%)	9 (90%)		14 (58%)	36 (57%)	
Year of surgery (*N*)						
Before 1998	3 (23%)	3 (30%)	0.784	12 (50%)	31 (49%)	0.76
Since 1998	10 (77%)	7 (70%)		12 (50%)	32 (51%)	
Chemotherapy (*N*)						
Yes	6 (46%)	4 (40%)	0.41	9 (38%)	16 (26%)	0.168
No	7 (54%)	6 (60%)		15 (63%)	47 (74%)	
Anti-hormonal treatment (*N*)						
Yes	8 (62%)	4 (40%)	0.522	13 (54%)	42 (67%)	0.203
No	5 (38%)	6 (60%)		10 (42%)	19 (30%)	
Unclear	0 (0%)	0 (0%)		1 (4%)	2 (3%)	
Tumor burden (%)						
Median	70	70	0.101	70	70	0.401
Range	50–90	50–90		50–90	50–90

**Table 2:** Clinical outcome of the patients analyzed for hsa-miR-3651

Clinical outcome *N* = 110				
Parameter	Pilot phase *N* = 23		Validation phase *N* = 87	
	Relapse *N* = 13	Control *N* = 10	Relapse *N* = 24	Control *N* = 63
Follow up (months)				
Median	139	133	125	142
Range	26–190	98–187	44–196	17–207
Cancer related deaths (*N*)	3 (23%)	0 (0%)	6 (25%)	0 (0%)
Local relapse	13 (100%)	0 (0%)	24 (100%)	0 (0%)
Distant metastasis	2 (15%)	0 (0%)	7 (29%)	0 (0%)

**Table 3:** Clinical and therapeutic parameters of the patients analyzed for FRMD3

Patient and treatment characteristics *N* = 41						
Parameters	Pilot phase *N* = 21			Validation phase *N* = 20		
	Relapse *N* = 13 (%)	Control *N* = 8 (%)	*p* value	Relapse *N* = 9 (%)	Control *N* = 11 (%)	*p* value
Patient characteristics						
Age at diagnosis (years)						
Median	57	62.5	0.336	57	56	0.295
Range	36–71	41–74		40–79	44–78	
Menopause (*N*)						
No	3 (23%)	1 (13%)	0.633	2 (22%)	4 (36%)	0.603
Yes	7 (54%)	5 (63%)		7 (78%)	7 (64%)	
Unclear	3 (23%)	2 (25%)		0 (0%)	0 (0%)	
T (*N*)						
T1	7 (54%)	2 (25%)	1	8 (89%)	9 (82%)	0.824
T2	6 (46%)	6 (75%)		1 (11%)	2 (18%)	
N (*N*)						
N0	11 (85%)	6 (75%)	0.595	7 (78%)	5 (45%)	0.412
N1	1 (8%)	1 (13%)		2 (22%)	6 (55%)	
N2	1 (8%)	1 (13%)		0 (0%)	0 (0%)	
M (*N*)						
M0	13 (100%)	8 (100%)	1	9 (100%)	11 (100%)	1
Grading (*N*)						
G1	0 (0%)	0 (0%)	0.804	2 (22%)	2 (18%)	0.941
G2	6 (46%)	4 (50%)		4 (44%)	6 (55%)	
G3	7 (54%)	4 (50%)		3 (33%)	3 (27%)	
Histology (*N*)						
IDC	11 (85%)	8 (100%)	0.804	6 (67%)	10 (91%)	0.37
ILC	2 (15%)	0 (0%)		1 (11%)	0 (0%)	
Other	0 (0%)	0 (0%)		2 (22%)	1 (9%)	
In situ component (*N*)						
Yes	12 (92%)	5 (63%)	0.268	3 (33%)	8 (73%)	0.152
No	1 (8%)	3 (38%)		6 (67%)	3 (27%)	
Receptors (*N*)						
ER positive	6 (46%)	4 (50%)	0.547	6 (67%)	9 (82%)	0.603
ER negative	7 (54%)	4 (50%)		3 (33%)	2 (18%)	
PR positive	7 (54%)	3 (38%)	0.5	6 (67%)	7 (64%)	0.941
PR negative	6 (46%)	5 (63%)		3 (33%)	4 (36%)	
her2neu (*N*)						
Positive	8 (62%)	1 (13%)	0.064	3 (33%)	4 (36%)	0.73
Negative	5 (38%)	7 (88%)		6 (67%)	5 (45%)	
Not assessable	0 (0%)	0 (0%)		0 (100%)	2 (18%)	
Proliferation index (*N*)						
ki67 < 20%	8 (62%)	3 (38%)	0.374	6 (67%)	6 (55%)	0.656
ki67 > 20%	5 (38%)	5 (63%)		3 (33%)	5 (45%)	
Not assessable	0 (0%)	0 (0%)		0 (0%)	0 (0%)	
Treatment						
Boost						
Intraoperative (*N*)	7 (54%)	4 (50%)	0.916	6 (67%)	7 (64%)	0.941
Percutaneous (*N*)	6 (46%)	4 (50%)		3 (33%)	4 (36%)	
None	0 (0%)	0 (0%)		0 (0%)	0 (0%)	
Intraoperative dose (Gy)	10	10		10	10	
Percutaneous dose (Gy)	12	12		12	12	
WBRT dose (Gy)						
Median	54	54	1	54	10	1
Range	52.2–55.6	54–56		51–59	10–12	
Surgery (*N*)						
BCT	13 (100%)	8 (100%)	1	9 (100%)	11 (100%)	1
Mastectomy	0 (0%)	0 (0%)		0 (0%)	0 (0%)	
Re-excision (*N*)						
Yes	5 (38%)	0 (0%)	0.645	2 (22%)	8 (73%)	0.056
No	8 (62%)	8 (100%)		7 (78%)	3 (27%)	
Year of surgery (*N*)						
Before 1998	3 (23%)	2 (25%)	1	3 (33%)	4 (36%)	0.941
Since 1998	10 (77%)	6 (75%)		6 (67%)	7 (64%)	
Chemotherapy (*N*)						
Yes	6 (46%)	3 (38%)	0.185	2 (22%)	2 (18%)	0.882
No	7 (54%)	5 (63%)		7 (78%)	9 (82%)	
Anti-hormonal treatment (*N*)						
Yes	8 (62%)	3 (38%)	0.75	6 (67%)	8 (73%)	0.824
No	5 (38%)	5 (63%)		2 (22%)	3 (27%)	
Unclear	0 (0%)	0 (0%)		1 (11%)	0 (0%)	
Tumor burden (%)						
Median	70	70	0.185	70	50	0.603
Range	50–90	50–90		50–90	50–90

**Table 4:** Clinical outcome of the patients analyzed for FRMD3

Clinical outcome *N* = 41				
Parameter	Pilot phase *N* = 21		Validation phase *N* = 20	
	Relapse *N* = 13	Control *N* = 8	Relapse *N* = 9	Control *N* = 11
Follow up (months)				
Median	139	133	110	124
Range	26–190	98–187	44–192	72–195
Cancer-related deaths (*N*)	3 (23%)	0 (0%)	2 (22%)	0 (0%)
Local relapse	13 (100%)	0 (0%)	9 (100%)	0 (0%)
Distant metastasis	2 (15%)	0 (0%)	2 (22%)	0 (0%)

The study was approved by the ethics committee of Salzburg (415-EP/73/85-2012 and 415-EP/73/582-2015). Informed consent was obtained from all individual participants included in the study.

### Samples

Tumor tissue from patients with early stage breast cancer retrieved during breast conserving surgery was formalin fixed paraffin embedded (FFPE) and stored in the tissue bank of the Department of Pathology. For the current analysis only samples with a minimum tumor content of 50% were eligible. Seven consecutive sections per patient were prepared. Isolation of total miR and chip-based microarrays (Agilent’s Sure PrintG3 Human miRNA microarrays™) were performed according to standard procedures by our external partner, the Comprehensive Biomarker Center™ (CBC), Heidelberg.

### MiRNA—pilot phase: microarray

The tumors of patients who experienced local relapse were compared to controls in order to screen for the most de-regulated microRNAs. The relative expression levels were calculated as fold change (relapse/control) with the ΔΔC_t_ comparative threshold method [23]. This part of the study was carried out by the external partner.

### MiRNA—validation phase: RT-qPCR procedure and data analysis

The most significantly de-regulated microRNA was tested in a separate cohort of 87 patients (24 relapses, 63 controls) by means of RT-qPCR. Briefly, small RNA molecules were extracted with miRNeasy FFPE kit (Qiagen™) and quantified fluorometrically with Qubit™ RNA Assay Kit (Invitrogen™). cDNA synthesis was performed with miScript II Kit (Qiagen™), and RT-qPCR analysis was done with miScript SYBR Green PCR Kit (Qiagen™) on Rotor-Gene (Qiagen™). A detailed description of each RT-qPCR step with respect to RNA extraction, reverse transcription, real time PCR and qPCR validation was previously published [7]. All samples were analyzed in duplicate reactions.

### Target proteins—pilot phase: microarray

A microarray for potential target proteins was carried out in the same FFPE samples that were used for the microRNA screen (“Patients” Section). Two patients had to be excluded for lack of biopsy material, which reduced the number of eligible specimen to 21 (13 relapses/8 controls). An in-silico search in six different databases (TargetScan, miRDB, PITA, DIANA, DIANA Cancer, accessed in May 2015; miRCarta accessed February 2021) was followed by a microarray (Affymetrix GeneChip Human Transcriptome Array 2.0^®^) containing 70,523 probes. From each sample, 5 nanogram total RNA was processed with the Affymetrix GeneChip WT Pico Reagent Kit^®^, which resulted in 5.5 µg ss-cDNA. Fragmenting, labelling and hybridization to the GeneChip® were performed according to the manufacturer’s instructions. The microarray was processed with the Gene Chip Hybridization Oven 645, Gene Chip Scanner and Gene Chip Fluidics Station 450 Dx (Affymetrix^®^, Thermo Fisher Scientific^®^). For image quality control the normalized unscaled standard error (NUSE) was used, which measures the accuracy of the expression data in relation with the other arrays in the batch [24].

### Target proteins validation phase

Tissue preparation was done according to the protocols provided by Promega^®^, while ddPCR and data analysis followed the BioRad^®^ manuals. A detailed description of the various steps of the target protein validation phase was previously published [8].

### Statistics

The microarray output data were analyzed with the robust machine learning algorithm (RMA) [25] from the oligo*-*package [26]. Differentially expressed microRNAs and target genes were selected as candidates for further validation according to their fold change and statistical significance estimated with the moderated t-test (linear models for micro-array analysis, LIMMA) [27]. Multiple testing was accounted for by the Benjamin–Hochberg correction method [28]. The Mann–Whitney test was used to compare ∆Ct-values, patient-, tumor- and treatment related characteristics between relapse and control groups. To generate a combined marker, the values for microRNA and target protein were ranked. Subsequently, these indices were subtracted from each other. Since an inverse relation between the microRNA and its target molecule could be assumed, the one-sided Pearson correlation test was used. Because of a reduced number of patients in the target validation phase the significance threshold was set at 0.2 for first order errors (α) in order to retain as much potentially predictive information as possible [29]. LC was estimated by the Kaplan–Meier-method with a log-rank comparison for subgroups.

## Results

### Patients

In the 23 patients that were screened for differential expression of microRNAs, potentially prognostic and predictive characteristics were evenly distributed between relapse and control groups (Table 1, pilot phase). The median follow-up in the pilot cohort (*N*  =  23) was 139 (range 26–190) and 133 months (range 98–187) for relapse and control patients, respectively (Table 2). In the validation cohort median follow-up was 125 (range 44–196) and 142 months (range 17–207) for relapse and control patients, respectively. In the relapse group of the pilot phase, two patients had distant metastases, occurring simultaneously with local recurrence in one case and eight months after local relapse in the other (Table 2). In the relapse group of the validation cohort, seven patients developed distant metastases, three of them at the time of local relapse, the other four at least 15 months after local relapse (Table 2). As for the target protein analysis, 41 patients were screened in the pilot phase (*N*  =  21) and validated in an independent set of patients (*N*  =  20). Again, potentially prognostic and predictive factors were evenly distributed between groups (Table 3). The median follow-up in the pilot phase was 139 (range 26–190) and 133 (98–187) months, respectively. In the validation phase the median follow-up amounted to 110 (range 44–192) and 124 (72–195) months, respectively. Three patients had metastases at the time of local relapse (two in the pilot and one in the validation phase), while one patient in the validation phase experienced isolated local relapse 24 months before distant progression (Table 4).

### MiRNA—pilot phase

**Fig. 1:**
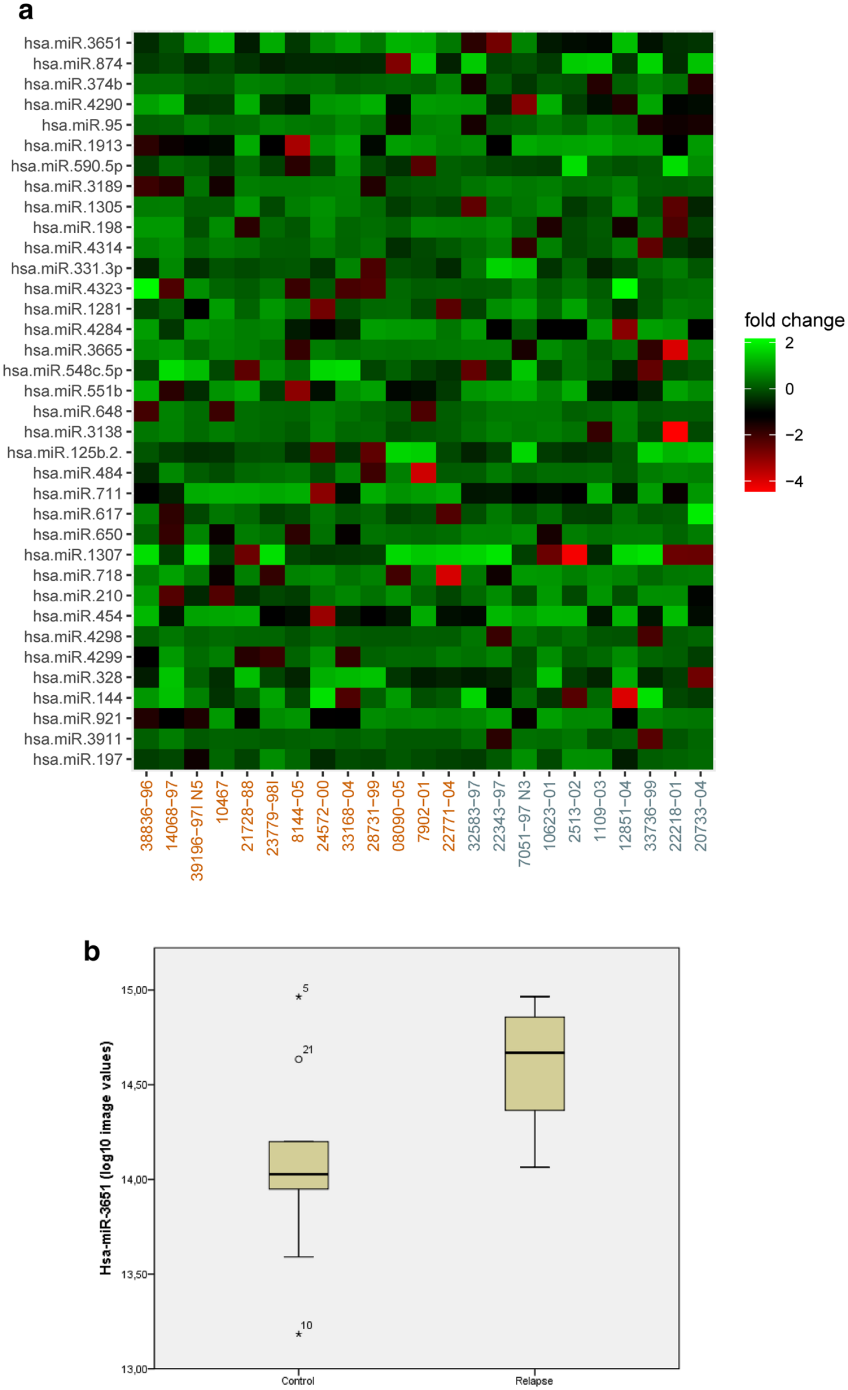
**a** Screen for de-regulated microRNAs: this heatmap shows the differentially expressed microRNAs with a raw *p *value  <  0.20 (LIMMA, *N*  = 23: 13 relapse patients and 10 controls). Hsa-miR-3651 has the highest statistical significance (relapse/control fold change  =  4.37; raw *p* value  =  0.0035). The fold changes (relapse/control) of a given miR are shown in green (high) and red (low). At the bottom the samples are shown: orange numbers refer to relapse patients, while controls are labeled blue. **b** Screen for de-regulated microRNAs: the median expression levels of hsa-miR-3651, image values from the microarray on the y-axis, were significantly higher in patients with local relapse compared to controls (*N*  =  23; Pearson correlation, *p *value  =  0.004)

The microarray revealed hsa-miR-3651 as the most differentially expressed miRNA (*N*  =  23, fold change 4.37; raw *p* value  = 0.0035; Fig. 1a, b). The array data were deposited in NCBI Gene Expression Omnibus (GEO): http://www.ncbi.nlm.nih.gov/geo/query/acc.cgi?acc=GSE69951.

### MiRNA—validation phase

**Fig. 2:**
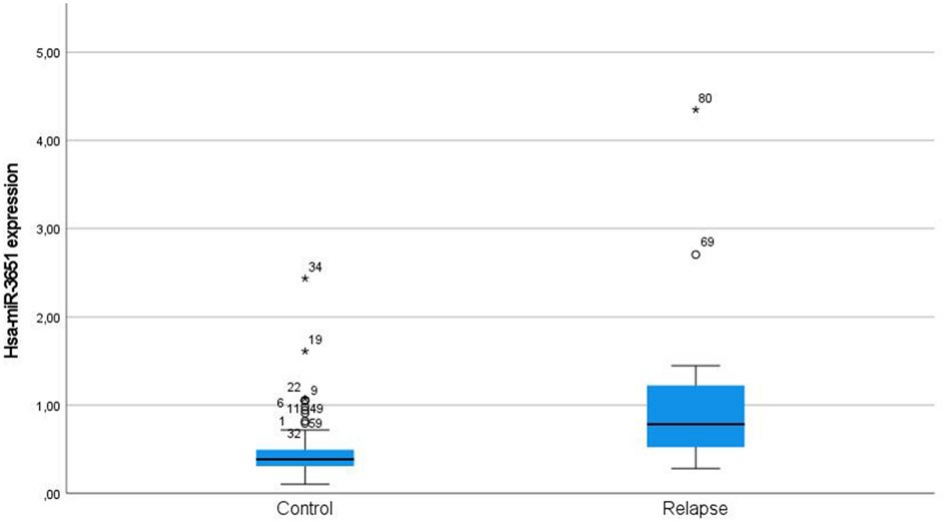
Validation of the most de-regulated candidate microRNA, i.e., hsa-miR-3651, by means of RT-qPCR. Hsa-miR-3651 expression levels in patients with local relapse were significantly higher than in controls (*N*  =  87, Mann–Whitney test, *p *value  <  0.0005). The fold change (relapse/control) was 2.86

**Fig. 3:**
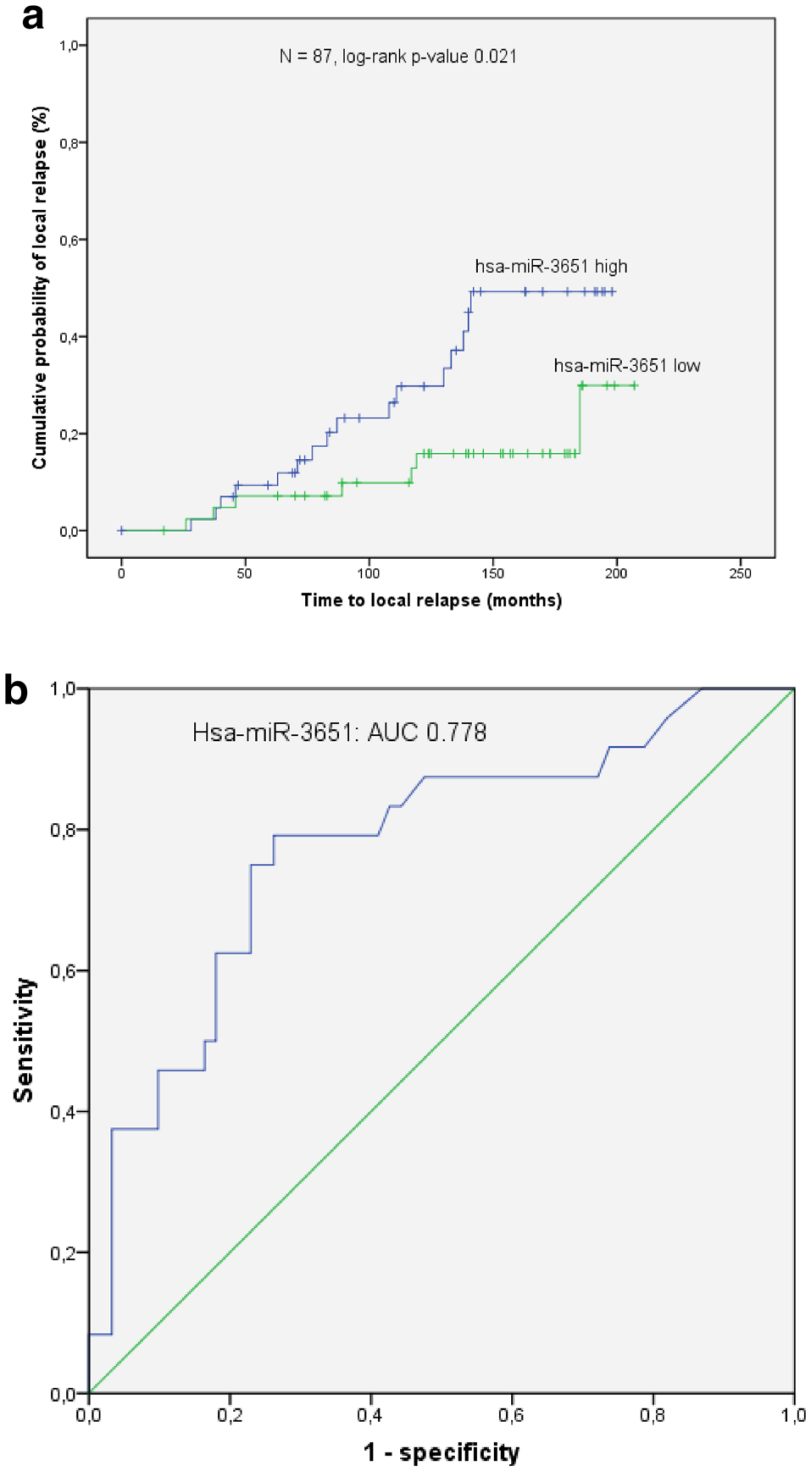
**a** In the time to event analysis (event  =  local relapse) patients with expression levels of hsa-miR-3651 above median (=  hsa-miR-3651 high) had a significantly higher probability for local relapse than those with low levels of the microRNA (*N*  =  87, log-rank *p *value  =  0.021). **b** The ROC analysis of hsa-miR-3651 revealed an AUC  =  0.778 (Mann–Whitney test; *p* value  <  0.0005)

The discriminatory potential with respect to LC was validated in an independent cohort. The levels of hsa-miR-3651 in these 87 patients were significantly elevated in the relapse group (*p* value  < 0.0005; fold change of 2.86; Fig. 2). In the time-to-event analysis high expression of hsa-miR-3651 correlated to a significantly enhanced risk of local relapse (*p* value  = 0.021; Fig. 3a). The corresponding ROC analysis revealed an AUC of 0.778 (*p* value  < 0.0005, Fig. 3b).

### Target proteins—pilot phase

**Fig. 4:**
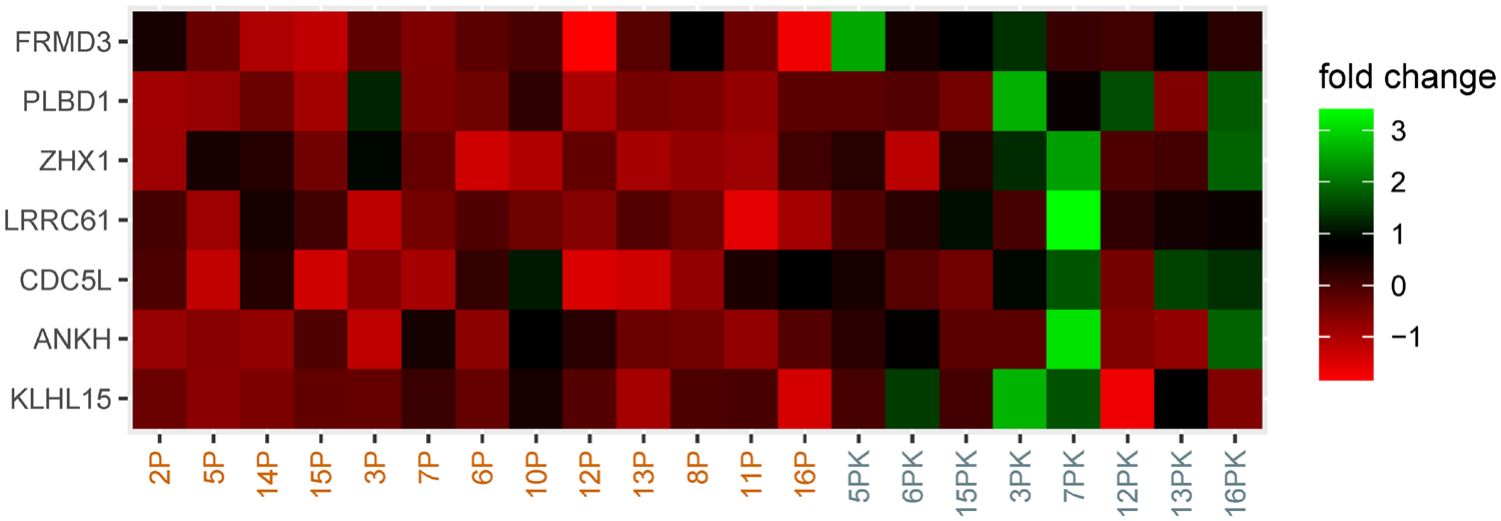
This heatmap shows the mRNA expression (fold change relapse/control) of seven target proteins that were down-regulated in relapse patients. FRMD3 was the most prominent molecule with a raw *p *value  = 0.0016 (Mann–Whitney test). The numbers at the bottom refer to relapse patients (orange) and controls (blue). The color bar at the right indicates the fold change: while green means relative over-expression, red signifies down-regulation

The patient and treatment characteristics of the 21 eligible revealed no significant differences between relapse and control groups (Table 3). The median follow-up was 139 (range: 26–190) and 133 months (range: 98–187) for relapse patients and controls, respectively (Table 4). Among the 168 potential target molecules, FRMD3 had a prediction score of 94.5% (miRCarta, accessed February 2021) with an 8mer binding site, which made it a plausible target candidate. This was corroborated by our screen (Fig. 4; supplementary file 2), which revealed FRMD3 as the most significantly down-regulated gene (*N*  =  21 patients, *p *value  =  0.0016). The array data were deposited in NCBI GEO (http://www.ncbi.nlm.nih.gov/geo/query/acc.cgi?acc=GSE156873). In this context, age deterioration plays an important role. Not surprisingly, this phenomenon could be observed in our analysis as well (supplementary file 2; Spearman correlation test, *p* value  = 0.021). Nevertheless it did not impact the comparison between relapse patients and controls, since the age distribution between the two groups was similar (supplementary file 3; Mann–Whitney test, *p *value  = 0.264).

### Target proteins—validation phase

**Fig. 5:**
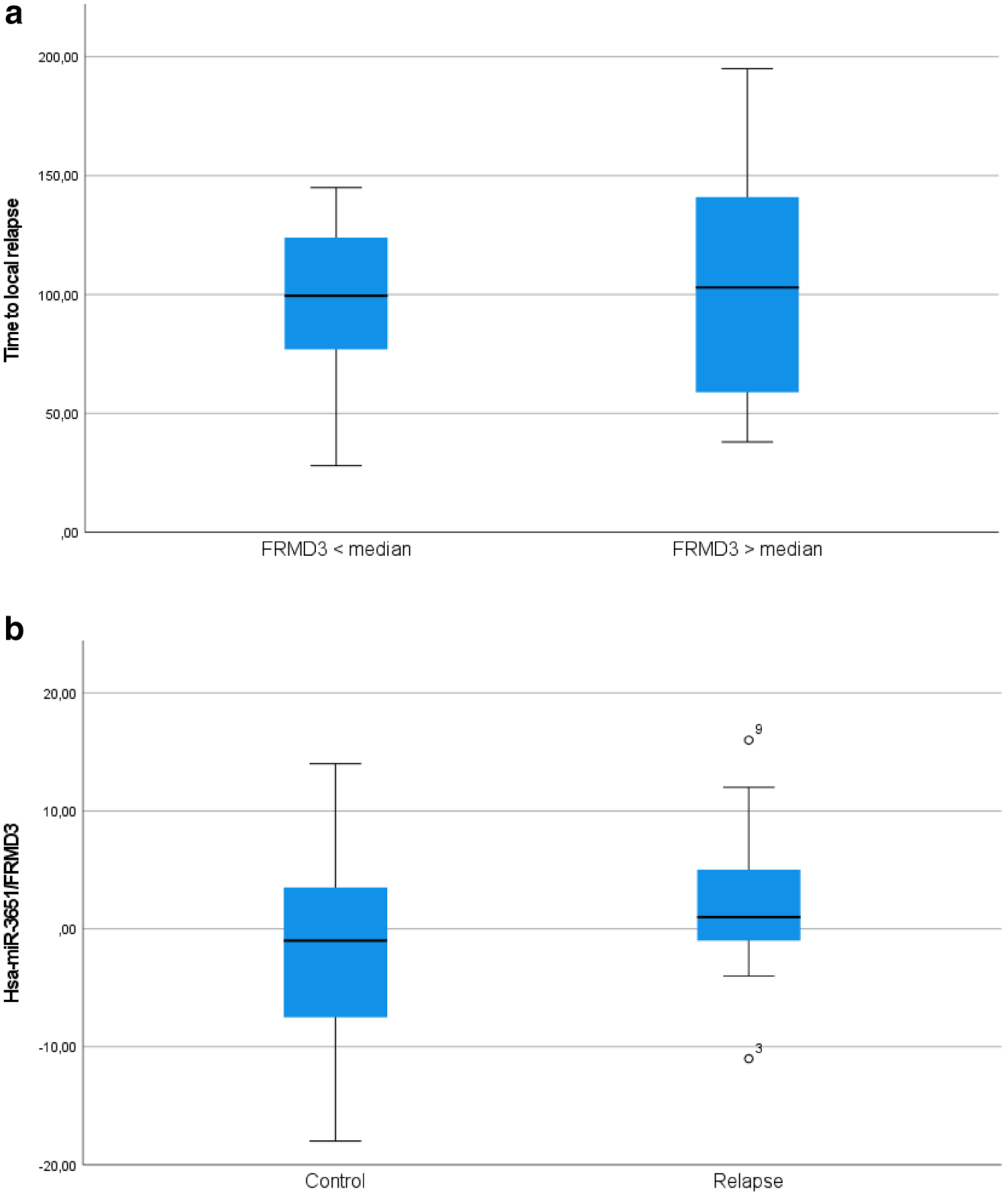
**a** Validation of the most prominently de-regulated target protein by means of ddPCR: patients with FRMD3 expression above median had longer time to local relapse (*N*  =  20; one-sided Pearson correlation, *p *value  =  0.164). **b** The correlation of the combined marker hsa-miR-3651/FRMD3 to local relapse revealed that the relapse group had a higher expression level of hsa-miR-3651 compared to FRMD3 (one-sided Pearson test, *p* value  =  0.134)

In an independent set of 20 individuals (9 relapses/11 controls), higher levels of FRMD3, i.e., above median, predicted longer latency to in-breast recurrence (*p* value  =  0.164, Fig. 5a). As for the combined marker hsa-miR-3651/FRMD3, a higher level of the microRNA compared to the target protein was observed in the relapse group (*p* value  = 0.134, Fig. 5b). This result was corroborated as a tendency in a time-to-event analysis (log-rank *p* value  = 0.260; supplementary file 5). Age dependent mRNA degradation was detectable in this part of the study, yet without influence on the results (supplementary file 7; Mann–Whitney test, *p *value  = 0.518).

## Discussion

The current analysis revealed hsa-miR-3651 as a novel predictor for LC in early breast cancer. Additionally, FRMD3 was identified as a putative target that interferes with protein binding as well as cytoskeletal and cell membrane stability.

While hsa-miR-3651 has been described as a discriminatory marker in other cancer types, such as head and neck [9, 10], ovaries [11], colorectal [12], liver [13], lung [14] and nephroblastoma [30], the current analysis is the first in breast cancer. From the viewpoint of sample selection, the study by Tuncer can be regarded as related to ours, in as far as it also investigates hormone dependent tissue. [11]. The authors analyzed microRNA profiles in peripheral blood lymphocytes of monozygotic twins with ovarian cancer and healthy relatives. They also found up-regulated hsa-miR-3651 in cancer patients [11]. A study in oral squamous cell carcinoma (OSCC) comparing healthy versus tumor tissue showed elevated levels of hsa-miR-3651 in the latter [9, 10] with a fold change of 2.5. Likewise, the authors of this study detected a correlation between hsa-miR-3651 and clinical features that indicate loco-regional invasiveness, such as lymph node stage. In contrast, Wang et al. showed that the down-regulation of hsa-miR-3651 in esophageal cancer patients led to worse OS and DFS [31]. The authors could also demonstrate a correlation between clinical factors that add to loco-regional aggressiveness, such as T-, N-stage and tumor length and the expression levels of hsa-miR-3651, implying that this micro-RNA may exert its (patho)physiological function in a tissue dependent manner [31].

As for the role of hsa-miR-3651 and its potential interference in functional pathways, FRMD3 was identified as a putative target by microarray technology (Fig. 4) and validated in an independent set of patient using ddPCR (Figs. 5; supplementary file 5). This molecule, which belongs to the protein 4.1 superfamily, is characterized by the highly conserved membrane-association domain FERM [15]. Although comprehensive analyses about its functioning are not available to date, it is generally assumed that it links cytoskeletal structures, i.e., actin filaments, to membrane proteins, which enhances cellular stability and impacts loco-regional invasiveness [16, 17, 32] (GeneCodis 4.0 accessed February 2021, supplementary file 6). Apart from its role in metabolic diseases [33–36], FRMD3 was reported to be focally expressed in hormone dependent tissue, i.e., adult ovary [37]. Additionally, in 58 NSCLC patient samples, Haase et al. found down-regulated FRMD3 in tumor compared to normal tissue [15]. In cell experiments the authors could show that it was able to induce apoptosis via the extrinsic, i.e., membrane dependent, pathway [15]. In contrast, reports on rectal [38, 39] and colon cancer [40] revealed that up-regulation of FRMD3 was associated with worse response to chemoradiotherapy. In two of the mentioned studies this molecule formed part of a multi-gene signature [39, 40]. In the third analysis, it was found to be the most up-regulated gene in nonresponders among 172 patients receiving cCRT [38]. Unfortunately, the authors did not verify their results in an independent set of individuals.

In summary, it can be said that in our study the de-regulation of hsa-miR-3651 measured as fold change is in the same range as in the above mentioned papers [9–11]. With due caution it can be assumed that this microRNA plays a role in loco-regional invasiveness [31], which is, at least to some extent, corroborated by the current study. In this context, FRMD3, identified as a potential target, might play a crucial role [16, 17]. Discrepancies with other studies can be explained by differences in the choice of clinical material and methodology. Our analysis was conducted in breast cancer, while published data originate from other tissues [9, 10, 31]. The current study compares primary tumor samples of patients with and without relapse, while other analyses used normal tissue specimens as controls. Since a major technical challenge consists in improving the ”signal to noise ratio”, only specimen with at least 50% tumor content were selected, which is comparable to published literature [20]. Other groups chose a higher cancer cell cutoff [6] or used micro-dissection [18, 19] in order to maximize the tumor signal. Finally, the interplay between different types of miRNAs is an issue, therefore we focused on single microRNAs. Since clustered miRNAs, which make up approximately 25% of the whole miRnome, are located in close genomic vicinity their transcriptional efficacy is presumably higher than that of single microRNAs [21]. Hence, it does not seem counter-intuitive to assume that the latter are underrepresented in whole miRNome investigations. To account for this potential loss of information, miRNA families were excluded in the current analysis.

In spite of a predicted 94.5% probability for the interaction between hsa-miR-3651 and FRMD3, no mechanistic evidence on a cellular level is extant thus far. Secondly, the direct measurement of FRMD3 protein levels, which would have certainly strengthened our results, was impossible both for lack of a suitable antibody and the small number of cases in the protein validation phase. Our results, however, are coherent with the two-fold physiological functioning of a microRNA, which results in decreased mRNA levels of a target protein either by RISC-mediated dissection of the mRNA molecule based on a perfect miRNA-mRNA binding or delayed degradation by less exact match. Hence, changes in mRNA levels can be regarded as an indirect measure for protein concentrations. Additionally, an 8mer miR-mRNA binding site allows to postulate a close affinity between hsa-miR-3651 and FRMD3. A general disadvantage of molecular analyses in samples of a certain age is the degradation of the tissue, which could also be observed in the current study (supplementary files 2, 3, 7).

## Conclusions

The current analysis demonstrates that hsa-miR-3651 may predict local control in early breast cancer via FRMD3. This hypothesis generating study provides a sound hypothesis to be tested in larger prospective studies, so that our results contribute to the framework of personalized medicine for this disease in the long run.

## Supplementary Information

Below is the link to the electronic supplementary material.

Supplementary file 1 (DOCX 633 KB)

## Data Availability

The array data were deposited in NCBI Gene Expression Omnibus under the accession numbers GSE69951 and GSE 156873, respectively; they can be downloaded via the following links: http://www.ncbi.nlm.nih.gov/geo/query/acc.cgi?acc=GSE69951, http://www.ncbi.nlm.nih.gov/geo/query/acc.cgi?acc=GSE156873.

## Abbreviations

AUC: Area under the curve
BCT: Breast conserving therapy
DdPCR: Droplet digital PCR
DFS: Disease free survival
EBCTG: Early Breast Cancer Trialist Group
FERM: Four-point-one, ezrin, radixin, moesin domain
FFPE: Formalin fixed paraffin embedded
FRMD3: FERM domain containing protein 3
LC: Local control
LIMMA: Linear models for micro-array analyses
miRNA, miR: MicroRNA
ncRNA: Non-coding RNA
NSCLC: Non-small cell lung cancer
OS: Overall survival
OSCC: Oral squamous cell carcinoma
PSVM: Potential support vector machine
RISC: RNA-induced silencing complex
RMA: Robust machine learning algorithms
ROC: Receiver operating curve
RT-Qpcr: Real-time quantitative polymerase chain reaction
3’UTR: Untranslated region
