# Supplementary material for: Hsa-miR-3651 could serve as a novel predictor for in-breast recurrence via FRMD3
Source: Breast Cancer. 2021 Dec 5;29(2):274–86. doi: 10.1007/s12282-021-01308-y (PMC8885475; doi:10.1007/s12282-021-01308-y)

Supplementary file 1. Experimental design


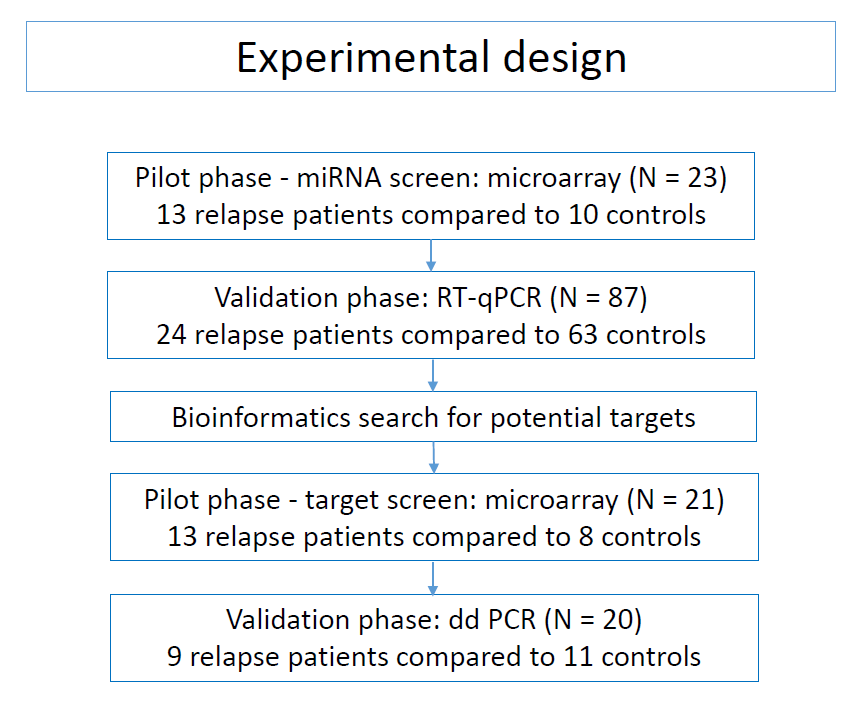


Supplementary file 2. This plot shows the correlation between NUSE and sample age. In fact, the oldest samples in the cohort (left) showed the highest NUSE levels (Spearman correlation test, p-value = 0.021).

Supplementary file 3. The comparison between relapse patients (P) and controls (PK) with respect to NUSE did not reveal a significant difference between the two groups (Mann-Whitney test, p-value = 0.264).

Supplementary file 4. The seven box plots show the predicted targets of FRMD3 that were down-regulated in relapse patients (p-value < 0.05), with FRMD3 showing the highest significance (p-value = 0.0016).

Supplementary file 5. Patients with a higher level of hsa-miR-3651 were more likely to experience local relapse although this difference did not reach statistical significance (log-rank p-value = 0.26).


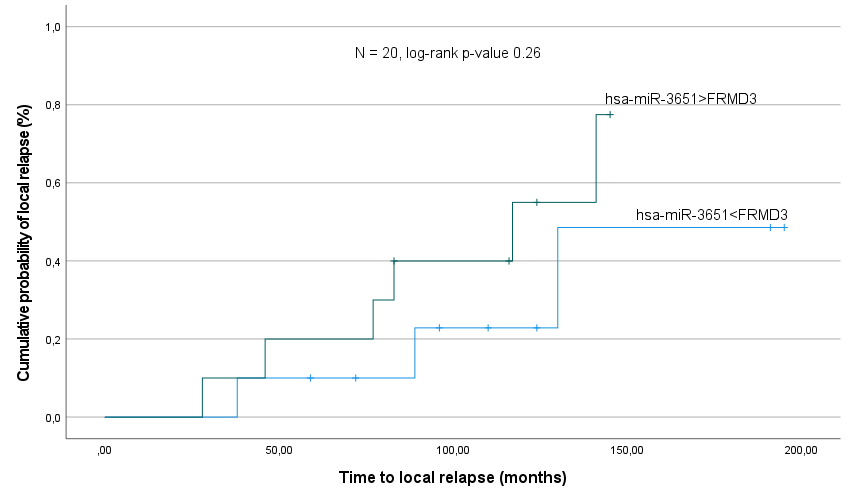


Supplementary file 6. These plots summarize the molecular function of FRMD3 (GeneCodis 4.0 accessed February 2021). FRMD3 seems to be involved in cytoskeletal protein binding, a role which it unfolds primarily in the membrane compartment.

1. Molecular function


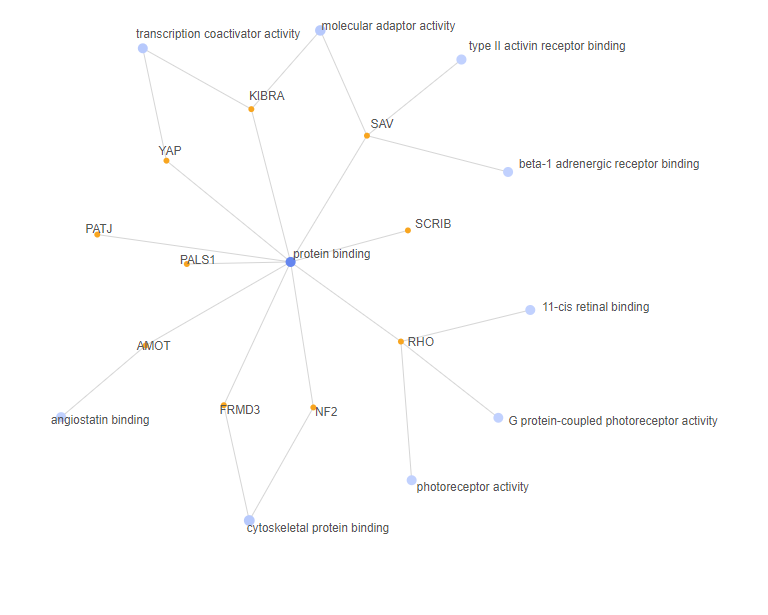


1. Cellular component


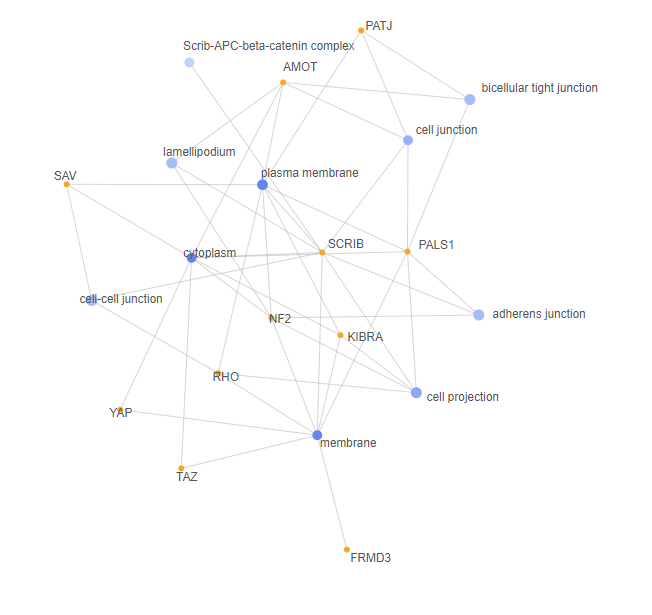


Supplementary file 7: To illustrate the effect of age on mRNA degradation, the ddPCR read-outs from two older (left) and younger (right) samples are contrasted with each other (Ntc = no template control). RNA extraction from the tissue blocks was performed simultaneously using 20 ng for PCR in two replicates. The number of positive droplets decreased with higher age (Mann-Whitney test, p-value = 0.073).


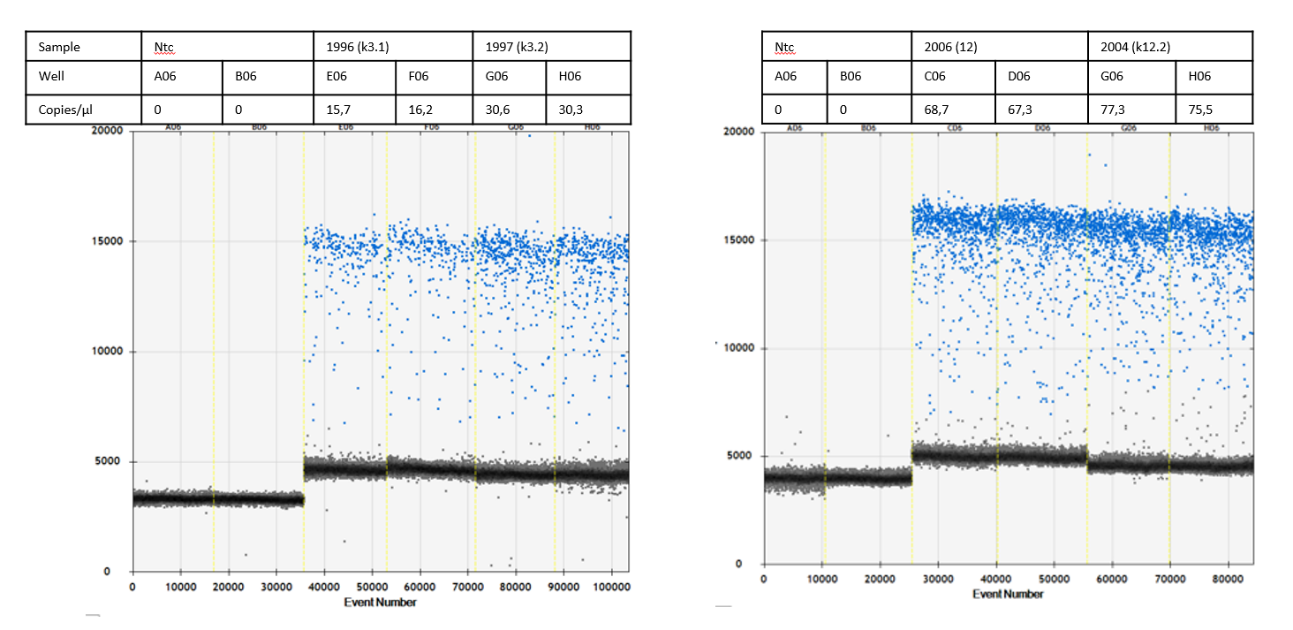

Supplement: Supplementary file 1 — (DOCX 633 KB) [file 12282_2021_1308_MOESM1_ESM.docx]
